# Endangered Right Whales Enhance Primary Productivity in the Bay of Fundy

**DOI:** 10.1371/journal.pone.0156553

**Published:** 2016-06-22

**Authors:** Joe Roman, John Nevins, Mark Altabet, Heather Koopman, James McCarthy

**Affiliations:** 1 Gund Institute for Ecological Economics, University of Vermont, Burlington, Vermont, United States of America; 2 Department of Organismic and Evolutionary Biology, Harvard University, Cambridge, Massachusetts, United States of America; 3 Department of Estuarine and Ocean Science, University of Massachusetts Dartmouth, Dartmouth, Massachusetts, United States of America; 4 Department of Biology and Marine Biology, University of North Carolina Wilmington, Wilmington, North Carolina, United States of America; Hawaii Pacific University, UNITED STATES

## Abstract

Marine mammals have recently been documented as important facilitators of rapid and efficient nutrient recycling in coastal and offshore waters. Whales enhance phytoplankton nutrition by releasing fecal plumes near the surface after feeding and by migrating from highly productive, high-latitude feeding areas to low-latitude nutrient-poor calving areas. In this study, we measured NH_4_^+^ and PO_4_^3-^ release rates from the feces of North Atlantic right whales (*Eubalaena glacialis*), a highly endangered baleen whale. Samples for this species were primarily collected by locating aggregations of whales in surface-active groups (SAGs), which typically consist of a central female surrounded by males competing for sexual activity. When freshly collected feces were incubated in seawater, high initial rates of N release were generally observed, which decreased to near zero within 24 hours of sampling, a pattern that is consistent with the active role of gut microflora on fecal particles. We estimate that at least 10% of particulate N in whale feces becomes available as NH_4_^+^ within 24 hours of defecation. Phosphorous was also abundant in fecal samples: initial release rates of PO_4_^3-^ were higher than for NH_4_^+^, yielding low N/P nutrient ratios over the course of our experiments. The rate of PO_4_^3-^ release was thus more than sufficient to preclude the possibility that nitrogenous nutrients supplied by whales would lead to phytoplankton production limited by P availability. Phytoplankton growth experiments indicated that NH_4_^+^ released from whale feces enhance productivity, as would be expected, with no evidence that fecal metabolites suppress growth. Although North Atlantic right whales are currently rare (approximately 450 individuals), they once numbered about 14,000 and likely played a substantial role in recycling nutrients in areas where they gathered to feed and mate. Even though the NH_4_^+^ released from fresh whale fecal material is a small fraction of total whale fecal nitrogen, and recognizing the fact that the additional nitrogen released in whale urine would be difficult to measure in a field study, the results of this study support the idea that the distinctive isotopic signature of the released NH_4_^+^ could be used to provide a conservative estimate of the contribution of the whale pump to primary productivity in coastal regions where whales congregate.

## Introduction

It is well established that microbes, zooplankton, and fish are important sources of recycled nitrogen and other nutrients in coastal waters [1, 2]. Recent studies have documented that whales and other air-breathing vertebrates also contribute to primary production through the vertical mixing, horizontal transfer, and recycling of limiting nutrients [3, 4]. Quantitative assessments of these mechanisms make it increasingly clear that marine mammals can provide an important ecosystem service by sustaining productivity in regions where they occur in high densities [5, 6]. Yet the relative importance of the great whales—a group that includes baleen whales and sperm whales—for nutrient recycling in the coastal ocean has been greatly reduced by human activities including commercial hunting and other activities that have increased nutrient release into this environment, such as sewage, atmospheric deposition, and agricultural runoff [4].

In this study, we examined the potential for North Atlantic right whales (*Eubalaena glacialis*) to pump nutrients to the euphotic zone for phytoplankton production in the Bay of Fundy, a tidally mixed basin. The right whale generally feeds on late-stage copepods (*Calanus finmarchicus*) during the summer and fall in this area [7, 8]. The bulk of the *C*. *finmarchicus* population is in diapause (stage V) at this time, remaining at depths below 100 m of the water column, with actively feeding copepods migrating vertically in the upper 100 m of the water column [7]. Right whales feed in high densities within a relatively well defined area of the bay, approximately 24 x 30 km [9] leaving visible orange-brown feces at the surface that can also be detected by smell [10]. Right whale fecal material is typically clumped, reaching lengths of up to 15 cm. It often floats at the surface, with sufficient cohesion to be collected intact using a dip net.

**Fig 1:**
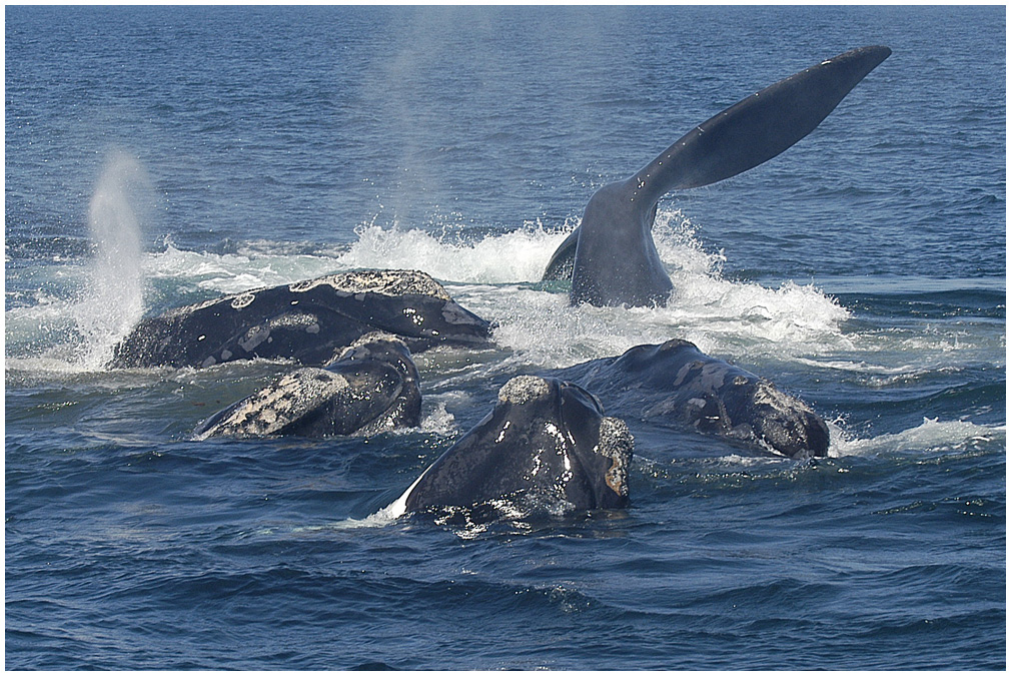
A surface-active group of at least five North Atlantic right whales in the Bay of Fundy. Fecal material is suspended in the surface water. Photo courtesy of New England Aquarium.

Surface-active groups, or SAGs, are the most commonly observed surface social behavior of North Atlantic right whales [11]. Typically characterized by a vocalizing, or focal, female, with one or more males socializing at the surface, SAGs present a unique opportunity for the production, distribution, and collection of feces for analysis (Fig 1) [12, 13]. These groups appear to be engaged in courtship, with the focal female swimming on her back, presumably to avoid copulation, and males jostling for this opportunity when she rolls over to breathe. SAGs can last for hours. In addition to these potentially conceptive groups, right whales commonly form SAGs throughout the year, in groups that could have other potential functions such as play, mating practice, or the maintenance of social bonds [13]. During this rigorous activity, feces can readily disassociate into fine particles from the turbulence caused by the interacting whales. Although feces can be observed at the surface while right whales are feeding, SAGs present a convenient opportunity for fecal collection and can function as “hotspots” for the release of nutrients in surface waters.

Several rorqual whales, distinguished by longitudinal folds of skin below their mouth, also occur in the region, including humpback (*Megaptera novaeangliae*), sei (*Balaenoptera borealis*), and fin (*Balaenoptera physalus*) whales. These rorquals feed on zooplankton (typically krill), schooling fish, and occasionally cephalopods. They release feces that can be clumped and positively buoyant when feeding on crustaceans, but tend to be more dispersed, and plume-like when they are consuming fish. These fine particles are readily dispersed by physical mixing.

To examine the ecological role of whales in the Bay of Fundy, we analyzed fecal samples from right whales, plus an occasional humpback, sei, and fin whale, for NH_4_^+^ and PO_4_^-3^ production rates and isotope ratios. From our previous work with humpback whales on Stellwagen Bank [4], we expected to find elevated levels of NH_4_^+^ associated with the right whale fecal material in the Bay of Fundy. Ammonium is of particular ecological significance since it is rapidly and efficiently utilized for phytoplankton growth, resulting in preferential uptake of this nitrogenous nutrient [14]. Phosphorus is essential for the structural and functional components of all organisms; recent studies have found high levels of P in whale feces [15], suggesting that whales may play an important role in distributing these nutrients to surface waters [16]. Micronutrients, such as iron and manganese, have also been found in whale feces [15], though we did not examine them here.

Over the past three decades, field and laboratory studies have revealed substantial new understandings related to isotopic fractionation, or the relative abundance of stable isotopes, in the marine nitrogen cycle; the application and utility of this approach in studies of marine ecosystems and biogeochemical cycles has been reviewed in detail [17, 18]. Analyses for δ^15^N of the fecal N and released NH_4_^+^ allowed us to explore the utility of this approach in assessing the contribution of right whales to nutrient availability in its feeding grounds.

## Materials and Methods

Field sampling was conducted between August 13 and September 2, 2011, under the aegis of the Grand Manan Whale and Seabird Research Station (GMWSRS), Grand Manan Island, New Brunswick, Canada. The research station provided laboratory facilities and an outboard motor boat for sample collection in nearby whale feeding areas.

### Sampling

**Fig 2:**
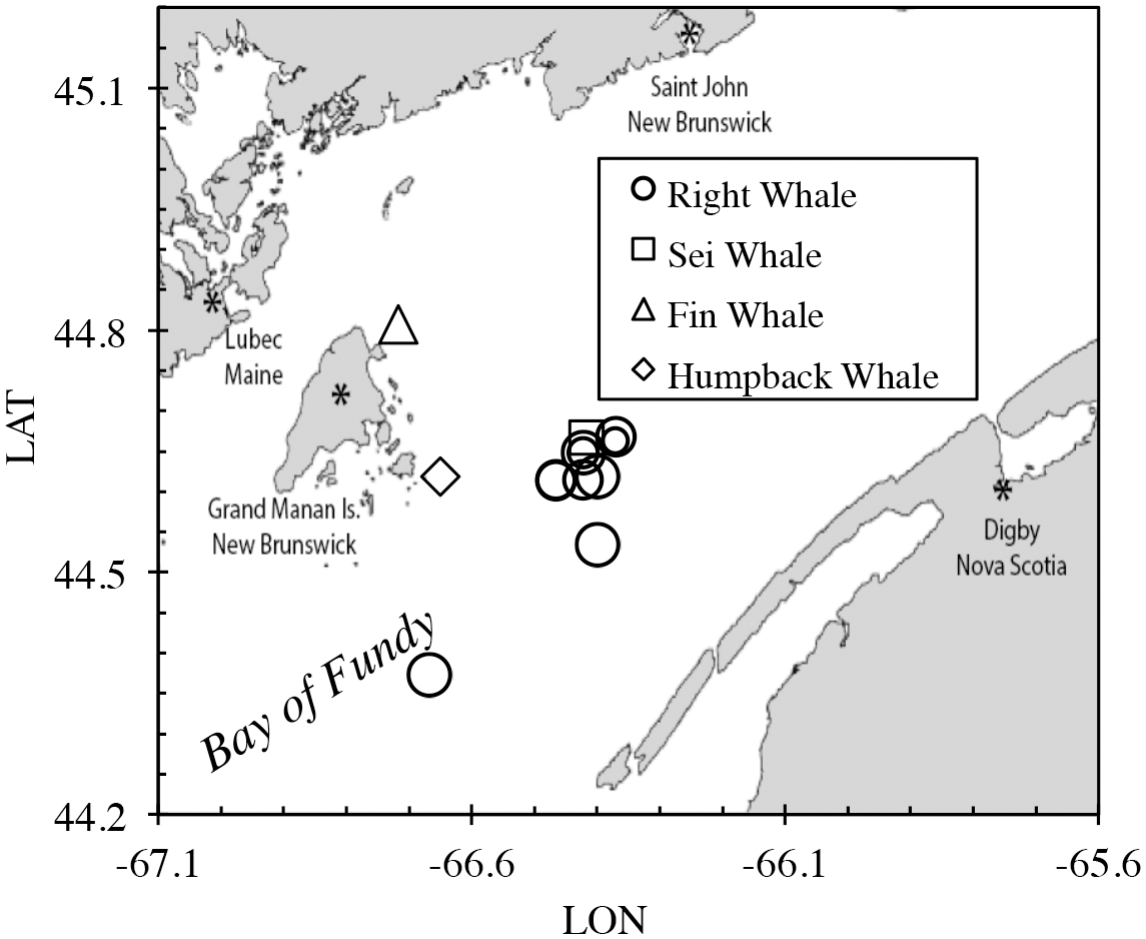
Whale fecal sampling locations in the Bay of Fundy. Sampling methods followed the protocols described by Roman and McCarthy [4].

Inshore waters in Grand Manan Basin (~200 m average depth) to the east of Grand Manan Island were sampled from a small boat (Fig 2). Right whales were located by running search patterns augmented by information sharing with researchers from the New England Aquarium (NEA) who were also working in this area. Whales were most often encountered in surface-active groups (SAGs of 5 to 25 individuals), which remained at or very near the sea surface for several hours at a time. Once located, the groups were approached to a distance of about 50 to 100 m, and as the group activity drifted away from its initial location, the boat moved into the area for water sampling and fecal material collection. Turbulence in these sample areas was visible for several minutes after the whales’ departure, and flocculent plumes and clumps of feces were captured. Given the depth of the water column in the area, which is typically more than 100 m, we do not expect that surface-active whales stirred up bottom sediment prior to our sampling.

We collected fecal suspensions with a 30-cm-diameter, 150-μm mesh plankton net attached to a 2-m pole. Samples were also collected at the waterline with a wide-mouth plastic bottle and in some cases by scooping globular feces from the surface, which were resuspended in surface seawater. Seawater control samples were collected several km away from recent whale sightings. All sample bottles were immediately placed in an ice chest containing frozen gel blocks and returned to the shore lab as soon as was practicable.

In addition to the right whale samples described above, feces of humpback, fin, and sei whales were collected by New England Aquarium and GMWSRS colleagues working nearby and using similar methods. Colleagues at the University of Alaska Southeast provided archived frozen fecal samples from humpback whales.

### Shore-lab processing of fecal suspensions

Fecal samples from the Bay of Fundy were processed at the Grand Manan Whale and Seabird Research Station as soon as they were brought to shore, generally between 5 and 7 hours from the time of collection. Samples were prepared for analyses of phytoplankton nutrient concentrations (NO_3_^-^, NH_4_^+^, and PO_4_^3-^), particulate organic nitrogen (PON) concentration, and N stable-isotope ratios for PON and NH_4_^+^. Frozen archived samples were resuspended in fresh ambient seawater collected away from whales and analyzed without incubation.

#### PON concentration and ^15^N natural abundance (δ^15^N)

Replicate aliquots of 10–50 ml (depending on visual assessment of particulate density in the sample) were filtered through combusted 25 mm Whatman GF/F glass fiber filters. The filters were then transferred to glass shell vials and placed in a 60°C drying oven for 24 h before capping and storing for later analysis of PON and δ^15^N on a Europa Scientific model 20/20 continuous flow, elemental analyzer/stable isotope ratio mass spectrometer system at Harvard University [19, 20].

#### δ^15^N NH_4_^+^ isotope ratio analysis

Aliquots were filtered through combusted 47mm GF/F filters to obtain 200ml of filtrate from each sample. NH_4_^+^ isotopic composition (δ^15^N) was measured according to a modified version of Zhang et al. [21]. Briefly, NH_4_^+^ is oxidized to nitrite using hypobromite and then reduced to N_2_O using acetic acid–buffered sodium azide. Isotope determinations were made at the University of Massachusetts Dartmouth using a GV IsoPrime IRMS, a custom purge-trap sample preparation system, and a CTC PAL autosampler. Reference materials used for calibration were IAEA-N1 and IAEA-N2 and values are reported relative to AIR with reproducibility better than ± 0.5%.

#### Nutrient analysis

Filtrate from each sample (~180 ml) was preserved by acidification to pH 2–3 with HCL for subsequent analysis of [NO_3_^-^], [NH_4_^+^] and [PO_4_^3-^] on a robotic Westco SmartChem^®^ 200 autoanalyzer (±0.05 to 0.2 μmol *l*^−1^ instrument precision depending on analyte), using standard colorimetric chemistries [22] at the Altabet Lab, UMass Dartmouth.

#### Incubation of fecal suspensions

After the initial samplings described above, the bottles containing fecal suspensions from the Bay of Fundy were placed in a dark temperature-controlled water bath, maintained at the temperature (±1°C) of the surface seawater measured at the collection sites. Samples were drawn at ~12 h intervals to obtain time courses of up to ~80 h from the incubated fecal suspensions and analyzed for particulate and dissolved constituents as described above.

#### Phytoplankton uptake of whale-derived fecal NH_4_^+^

Nitrogen-uptake experiments [23] were conducted to determine if NH_4_^+^ released from whale feces would enhance phytoplankton growth. Ambient surface seawater was collected in the Bay of Fundy at a distance of more than 1 km from recent whale activity (ambient NH_4_^+^ </ = 0.05 μmol kg^-^). Two 1-*l* aliquots of the single surface seawater sample were enriched to 8 μmol kg^-1^ NH_4_^+^ with right whale fecal matter filtrate from samples F1 and F13. A third aliquot without this addition was used as the untreated control. Since the NH_4_^+^ concentration was substantially elevated in the experiments with filtrate addition, we expected that the concentration of NH_4_^+^ would decline and the concentration of PON would rise relative to the control during the course of the experiment. Moreover, since the δ^15^N of the added NH_4_^+^ was substantially higher (~12‰) than the initial δ^15^N PON (~6‰), uptake of NH_4_^+^ into PON would result in an increase in δ^15^N PON. An increase in chlorophyll *a* (Chl *a*) commensurate with an increase in PON would allow us to infer that the uptake was from phytoplankton assimilation. Initial PON concentrations and δ^15^N were determined by filtering an aliquot from a control sample onto a GF/F filter as described above. A second aliquot was filtered and kept frozen for subsequent Chl *a* analysis at Harvard University [22]. (The concentration of Chl *a* was used as a proxy for phytoplankton biomass. No data are available for phytoplankton species abundances.)

The samples were incubated for 9.5 hours, beginning in midmorning, in a water bath under simulated in situ conditions [20, 24] at 17°C (±1) and with 36% ambient sunlight. The incubations were terminated by filtration, and the filters were dried and stored for later analysis.

## Results

**Fig 3:**
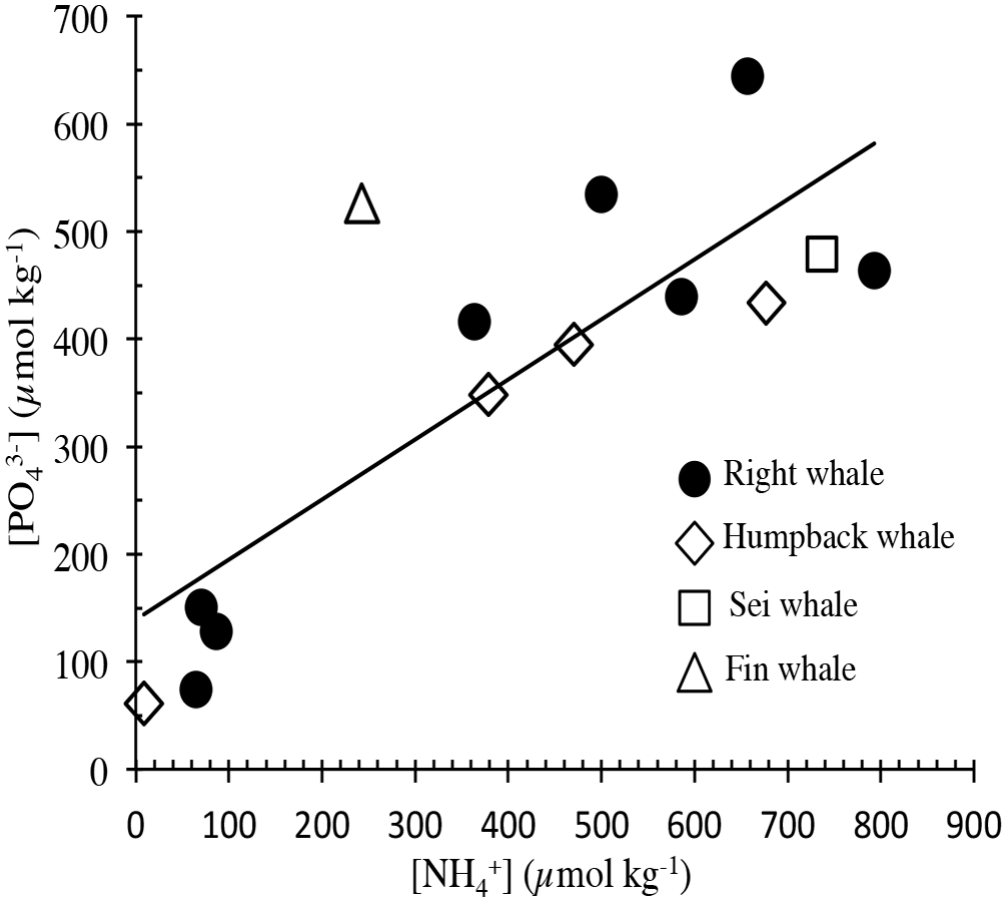
Regression analysis of dissolved NH_4_^+^ and PO_4_^3-^ concentrations in whale feces filtrate at initial sampling. For all species combined, R^2^ = 0.73.

**Fig 4:**
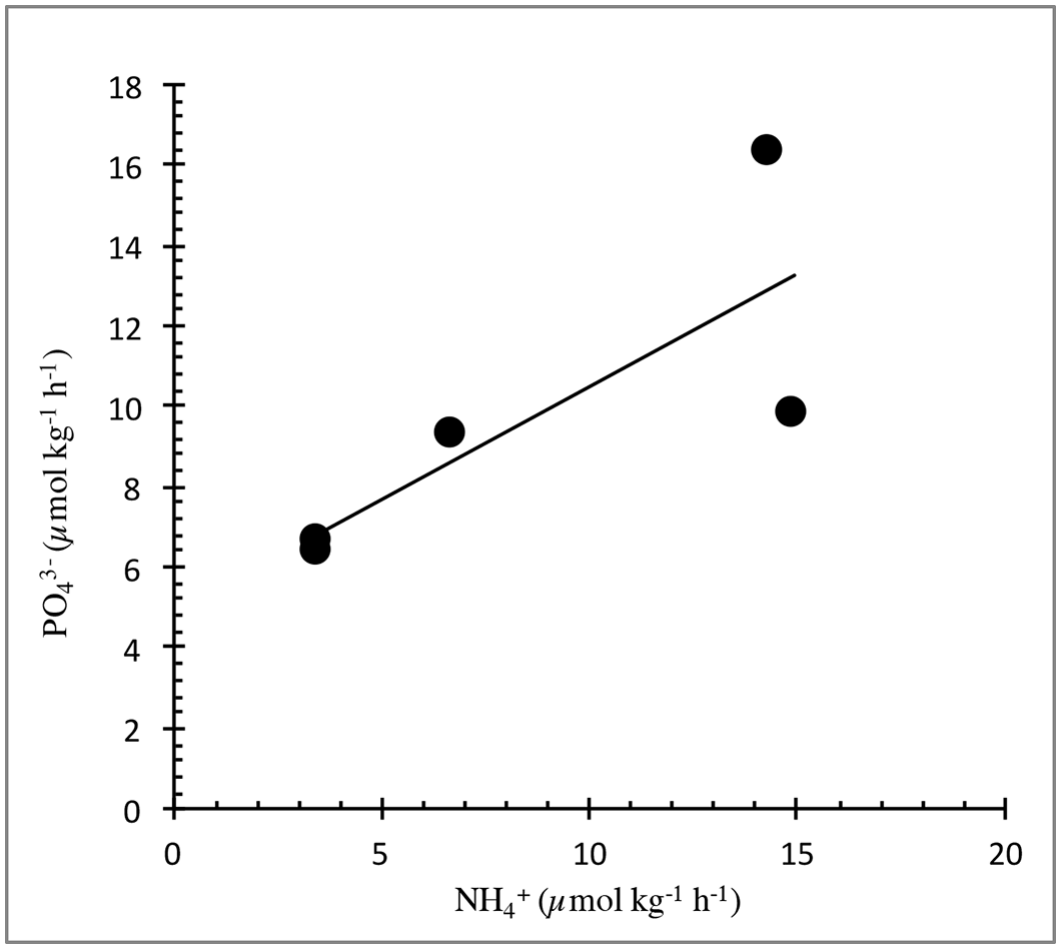
Regression analysis of release rates for NH_4_^+^ and PO_4_^3-^ for right whale feces. Rates were calculated for the period between the initial sampling and the first time point during the fecal suspension incubation experiments, R^2^ = 0.63.

Dates, locations, and analytical results for samples collected in 2011, including concentrations of dissolved and particulate constituents of the whale fecal suspensions, are presented in Table 1. Values of δ^15^N for PON and NH_4_^+^ are similar across the range of right whale feces and the three additional species sampled. Concentrations of NH_4_^+^ and PO_4_^-3^ were orders of magnitude higher than typical values for coastal waters [25–27]. Dissolved NH_4_^+^:PO_4_^-3^ molar ratios in the right whale fecal suspensions at the initiation of the incubation experiments (Table 1) are quite constrained with a mean of 0.7 (Fig 3). Data for PON and δ^15^N in right and humpback fecal samples provided by the New England Aquarium and the University of Alaska Southeast are presented in Table 2. The four points clustered near the origin in Fig 3 all have higher PO_4_^-3^ than NH_4_^+^ concentrations. Given typical Redfield ratios of ~16 N/P for marine plankton and detritus, it is likely that the high PO_4_^-3^ concentrations at the initial sampling point reflect a more rapid release for P than for N from the whale fecal material during the time between defecation and the initiation of the incubation experiments on shore. Evidence for this can be seen in the rates of release for NH_4_^+^ and PO_4_^-3^ between the first and second sampling points during the right whale fecal suspension incubation experiments (Fig 4, showing samples with increased NH_4_^+^). For all but one datum point, the release rate for PO_4_^-3^ during this interval exceeded that for NH_4_^+^.

**Table 1:** Whale fecal material sampling details and analytical results for dissolved nutrients, PON concentration, and N stable isotope ratios for PON and NH_4_^+^ at the first time point, 5–7 h after sampling.

Sample ID	Date	Lon (deg)	Lat (deg)	[PON] (μMol kg^-1^)	[NO_3_^-^] (μMol kg^-1^)	[PO_4_^3-^] (μMol kg^-1^)	[NH_4_^+^] (μMol kg^-1^)	δ^15^N PON (‰)	δ^15^N NH_4_^+^ (‰)
F1-RW	13 Aug 11	-66.371	44.663	1161	1.91	151	70	9.5	12.3
F5-RW	13 Aug-11	-66.370	44.668	206	0.32	440	586	9.6	13.1
F8-RW	23 Aug 11	-66.422	44.648	2186	5.34	644	657	7.9	17.3
F11-RW	24-Aug-11	-66.465	44.615	648	0.78	416	364	9.7	13.4
F12-RW	24-Aug-11	-66.465	44.614	1489	0.79	534	500	9.4	13.7
F13-RW	24-Aug-11	-66.399	44.534	547	0.90	128	86	8.8	12.4
F14-RW	24-Aug-11	-66.399	44.618	218	0.62	74	65	8.4	13.6
F15-RW	30-Aug-11	-66.668	44.373	1371	0.92	*	316	9.3	16.1
F16-RW	2-Sep-11	-66.422	44.648	348	5.24	463	793	9.5	13.1
							**Mean**	**9.1**	**13.9**
							**SD**	**0.62**	**1.6**
F7-HB	17-Aug-11	-66.649	44.663	146	6.13	61	8.8	9.0	9.0
F6-FW	13-Aug-11	-66.717	44.807	856	1.01	526	67	7.1	15.7
F18-SW	18-Sep-11	-66.417	44.667	416	3.59	479	737	9.8	12.0

RW is right whale, FW fin whale, HB humpback, SW sei whale.

* for no data.

**Table 2:** N analyses on frozen archived samples provided by colleagues from the New England Aquarium (NEA), and the University of Alaska, Sitka (UAS). BOF is Bay of Fundy; SeC Seymour Canal, AK; SiS Sitka Sound, AK;

Sample ID	Source		Locale	Date	Lon* (deg)	Lat* (deg)	[PON] (μMol kg^-1^)	N %	δ^15^N PON (‰)
RW1-08		NEA	BOF	8/08*	-66.7	44.4	146	6.2	13.4
RW2-08		NEA	BOF	8/08*	-66.7	44.4	856	7.5	12.5
RW3-08		NEA	BOF	8/08*	-66.7	44.4	416	8.0	†
F-19-HB		UAS	SeC	10/12/08	-134.1	57.8	326	†	9.5
F20-HB		UAS	SeC	11/15/08	-134.1	57.8	333	3.5	9.4
F-21-HB		UAS	SiS	6/22/08	-135.6	56.9	550	†	9.6

*approx. date or location;

^†^ no data.

**Fig 5:**
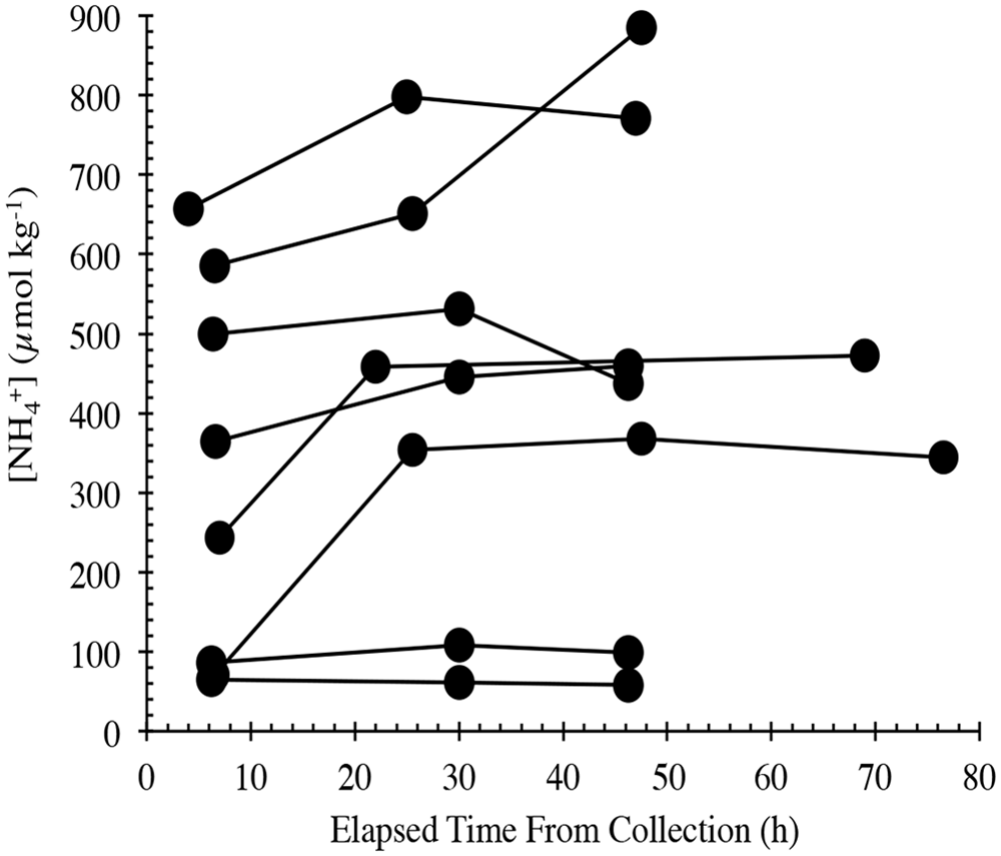
Time courses for NH_4_^+^ concentration in fecal suspensions from the Bay of Fundy right whale.

**Fig 6:**
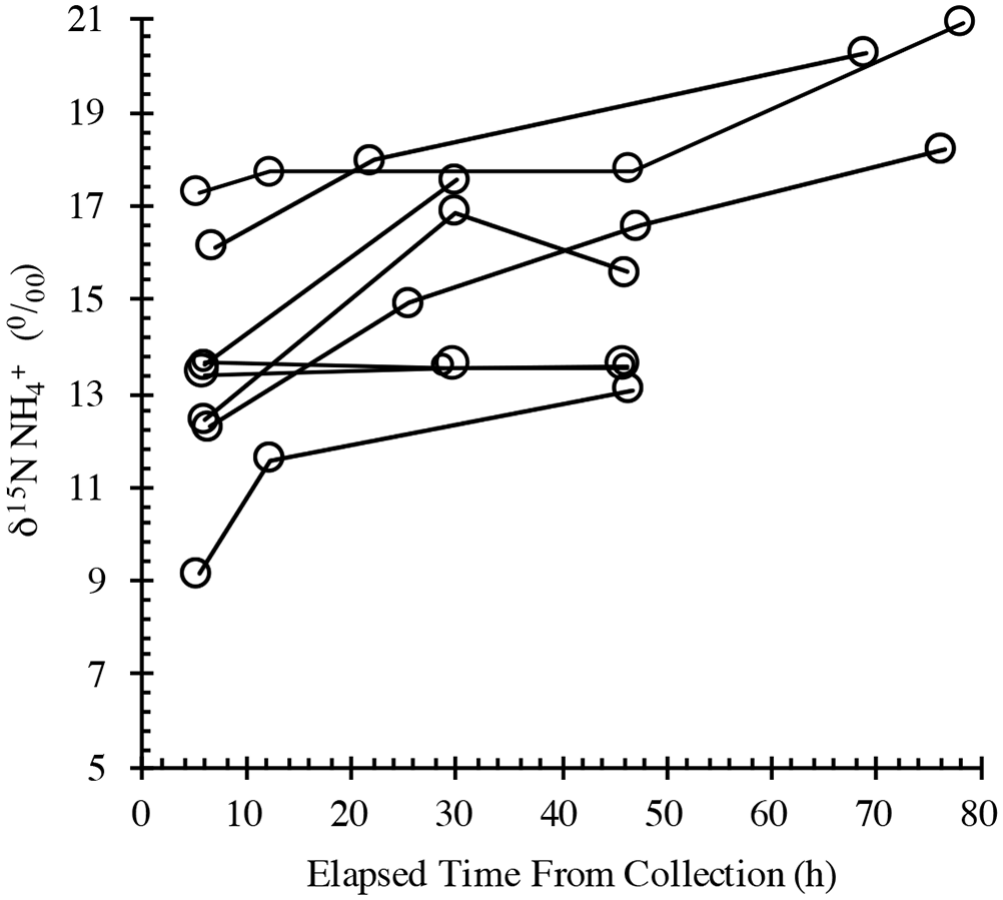
Time courses of δ^15^N NH_4_^+^ in fecal suspensions from the Bay of Fundy right whale.

The NH_4_^+^ release experiments in this study were run longer than the ~24h time period used by Roman and McCarthy [4], who found that NH_4_^+^ generally increased to the end point of the incubation. To determine whether these release rates were sustained over a longer period, we extended the time courses to > 48h. Ammonium was again observed to increase in the whale fecal suspensions during the first 24h of incubation, but with only a few exceptions, NH_4_^+^ either leveled off or declined beyond 30 hours (Fig 5). The time course for δ^15^N of right whale fecal PON and the NH_4_^+^ that was released during the incubation experiments is shown in Fig 6. In comparison with the NH_4_^+^ concentration data, the NH_4_^+^ δ^15^N data trended toward higher values in more of the experiments after the 24-hr time point.

Nutrient analyses on the filtrates from the whale-fecal incubations yielded undetectable concentrations of NO_2_^-^, and the concentrations of NO_3_^-^ ranged from about 1 to 5 μmol kg^-1^, which are typical for the surface waters of the central basin during late summer [25–27]. Subsequent samplings from the same whale fecal incubations used for the NH_4_^+^ and PO_4_^3-^ release experiments revealed no measureable increase in NO_2_^-^, or NO_3_^-^ concentrations (data not shown).

**Fig 7:**
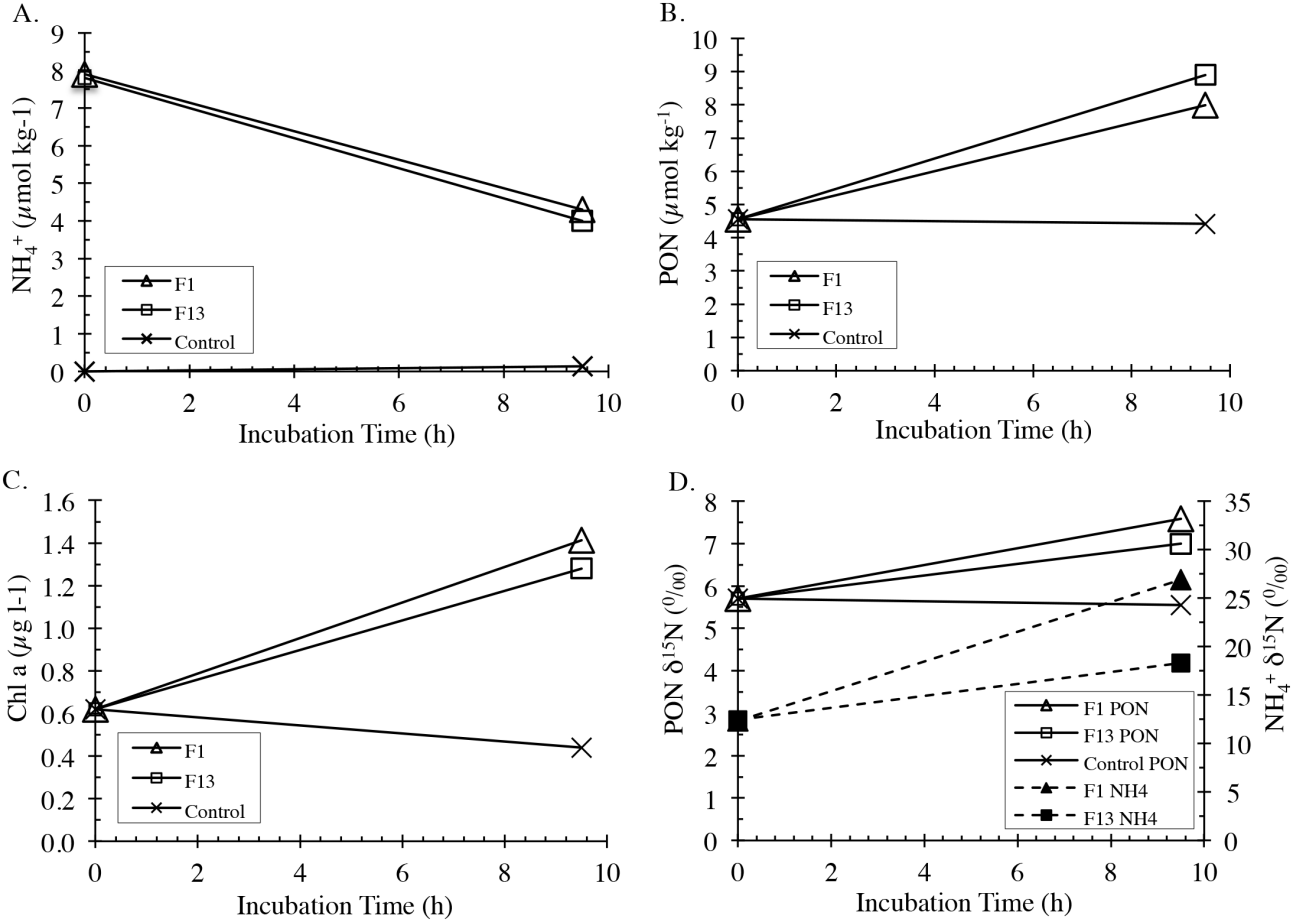
Uptake experiment using natural ^15^NH_4_^+^ tracer obtained from right whale fecal matter suspension filtrates. Change in NH_4_^+^ concentration over incubation time (a); change in PON (b); change in Chl *a* (c), changes in δ^15^N for NH_4_^+^ and PON (d).

In the uptake studies, it was evident that NH_4_^+^ derived from whale feces could support phytoplankton growth unhindered to any obvious degree by other metabolites associated with the fecal material (Fig 7). Assimilation of NH_4_^+^ (and likely PO_4_^3-^, though these rates were not measured) produced from this feces resulted in an approximate doubling of phytoplankton biomass as measured by PON and Chl *a* levels during the 10-hr experiment. (The control sample received no nutrient augmentation, and its Chl *a* concentration declined during the incubation.) As would be expected in a nutrient-enrichment experiment, the decrease in NH_4_^+^ concentrations, from about 8 to 4 μmol kg^-1^, approximately matched an increase in PON concentrations from about 4.5 to 8.5 μmol kg^-1^. Similarly, the Chl *a* concentration doubled from 0.6 to 1.3 μg l^-1^. These trends and the similar ratios of PON to Chl *a* concentrations, 6–7 μmol PON kg^-1^ per μg Chl *a* l^-1^, at the initiation and conclusion of this experiment, together suggest that the suspended PON consisted mostly of phytoplankton. The δ^15^N of PON increased as would be expected with uptake of the more highly ^15^N-enriched NH_4_^+^ derived from whale feces. The δ^15^N of residual NH_4_^+^ also increased, an expected result of the isotope fractionation associated with the preferential assimilation of the lighter N during phytoplankton uptake.

## Discussion

**Fig 8:**
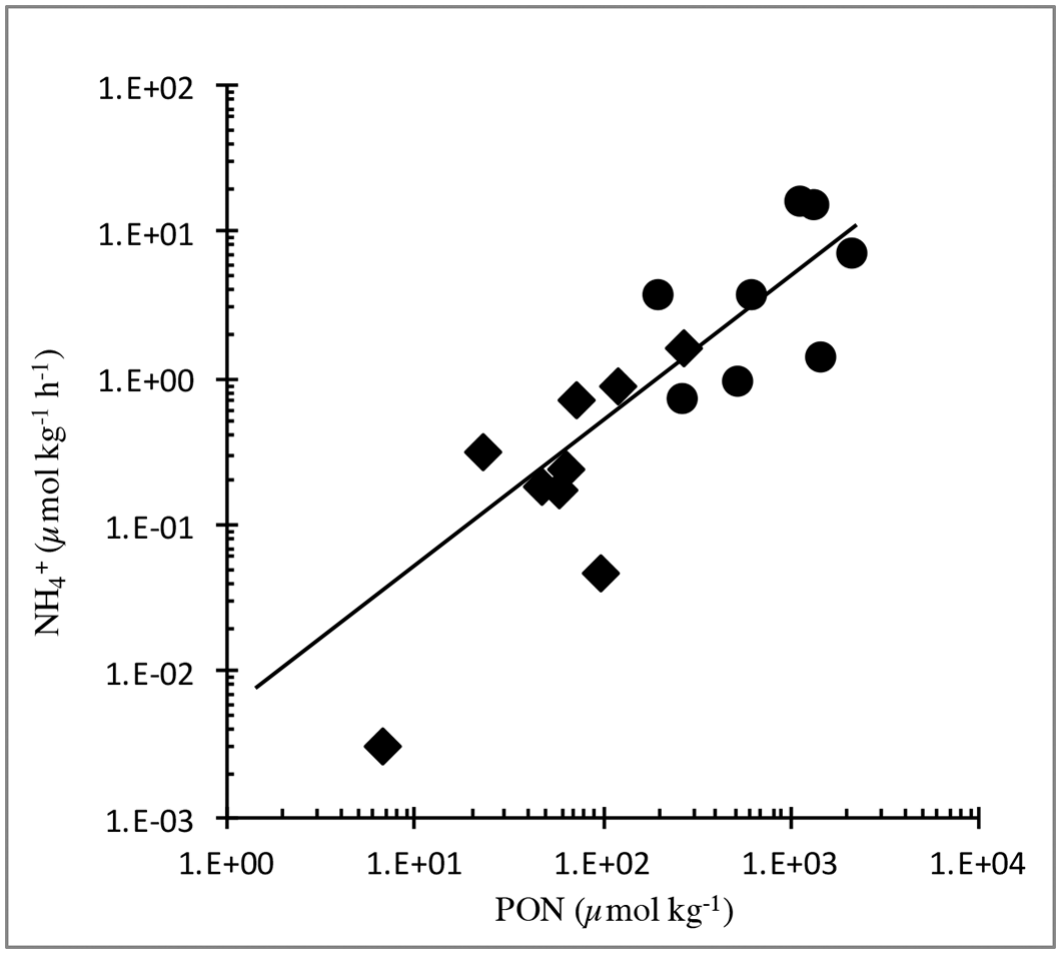
Rates of NH_4_^+^released vs. PON concentration for fecal suspensions. Data for initial ~20h of incubation, combining results for right whales (circles •, this study) and published data for humpback whales on Stellwagen Bank (diamonds ◆) [4].

Though long overlooked, the effect of marine mammals and other top predators on primary productivity and phytoplankton ecology is an emerging area of research [6, 15, 28]. It is interesting to note that both in our prior study, using humpback whales feeding on sand lance in Massachusetts Bay, and in this one, with right whales feeding on copepods, the yield of NH_4_^+^ as a function of fecal nitrogen concentration in our experiments is similar: ~4–5 nmol N (μmol fecal N)^-1^ hr^-1^. In other words about ten percent of the nitrogen in fresh feces is released as NH_4_^+^ day^-1^ (Fig 8). We would expect this NH_4_^+^ to diffuse and assimilate rapidly into the planktonic community of the euphotic zone. Whales can maintain prey aggregations by increasing nutrients in feeding areas when concentrations decline [4].

Right whale fecal material was relatively ^15^N enriched with average δ^15^N values of ~ 9‰ (s.d = 0.6, n = 9; Table 1) as compared to the oceanic average near 5‰ [29] and surface PON observed in this study (~5.8‰; Fig 7d). This enrichment is likely due to whale feeding at higher trophic levels relative to phytoplankton as δ^15^N increases by 3 to 4‰ per trophic step [30]. Even considering, though the data are few, other whale species consuming different food in different regions, this degree of enrichment appears to hold (Table 1). It follows that NH_4_^+^ released from whale fecal material would be ^15^N enriched as confirmed by average values for the first time point of the release experiments of ~14‰ (s.d. = 1.6‰). Further ^15^N enrichment in the released NH_4_^+^, however, was not expected as fractionation results in isotope depletion of the product. Instead it would appear that NH_4_^+^ is preferentially released from components of the fecal material with higher than average δ^15^N for this material. Increasing δ^15^N for released NH_4_^+^ during incubation (Fig 6) suggests this process intensifies with time.

Regardless of its cause, the high δ^15^N of released NH_4_^+^ provides a strong signal for natural isotope tracing of the importance of this N source for phytoplankton. Qualitatively, uptake of ^15^N enriched NH_4_^+^ readily accounts for the ~2‰ increase in PON in δ^15^N during the course of the incubation experiment (Fig 7d). Mass balance calculation shows that the added PON had a δ^15^N of ~ 9‰ similar to the average δ^15^N of NH_4_^+^ for the first time point of the release experiments (Table 1). However, this value is about 2‰ lower than the initial δ^15^N NH_4_^+^ in the incubation experiments, a likely consequence of isotope fractionation during phytoplankton uptake [31]. Such fractionation is consistent with the increase in δ^15^N NH_4_^+^ as its concentration is drawn down during the incubation. Beyond confirming that NH_4_^+^ release from whale fecal material can support phytoplankton growth in controlled experiments, these results prove the feasibility of using this isotope signal in field studies designed to ascertain the contribution of nitrogen supplied from whale feces to local phytoplankton productivity.

Our findings support a previous study in the Southern Hemisphere showing high concentrations of phosphorous relative to carbon in whale feces [15] and expand upon this work in documenting high rates of PO_4_^3-^ release during incubations with whale fecal material. It is possible that other soluble bioactive constituents in these samples—such as Fe, other trace metals, or organic compounds—could have stimulated phytoplankton activity. Pygmy blue whale feces have been found to increase the photosynthetic rate and growth of three marine phytoplankton species [32]. In that study, feces were air dried and crushed into a fine powder before their use, whereas our study used filtrate from right whale fecal matter. As with the previous study, it is not surprising that added nutrients and micronutrients would stimulate phytoplankton growth. Just as important, however, we found no evidence that fecal metabolites inhibited growth.

Phytoplankton Chl *a* content can change in response to irradiance level, but the light used for these incubations (36% surface light) is unlikely to have promoted a strong adaptive response during the experiment. Likely explanations for the ~30% decline in Chl *a* in the control bottle include cell lyses and consumption by small microzooplankton grazers [33]. Although no copepods or other macroplankton were visibly observed in any of the bottles, no effort was made to physically exclude grazers from these incubation bottles. If microzooplankton grazing did cause a reduction of phytoplankton in the control experiments, we would also expect that they would have consumed Chl *a* in the enriched experiments. Excluding all microzooplankton from such experiments is impractical. Future experiments like these, however, could estimate grazing by microzooplankton using the Landry dilution technique [34].

Chl *a* effectively doubled in the augmented experimental bottle, or more than doubled when compared to the control bottle. The decrease in NH_4_^+^ during the incubation approximately balances the increase in PON, which would be consistent with assimilation of NH_4_^+^ by phytoplankton and retention in the particulate pool. Analyses were not made for PO_4_^3-^ or particulate organic phosphorous, but it is likely that the concentrations of dissolved and particulate phosphorus changed relative to one another, and consistent with phytoplankton assimilation, in a manner similar to nitrogen.

Filtration of the whale fecal material (GFF filter, nominal porosity ~0.7 μm) for this enrichment experiment would not have removed bacterioplankton. Thus, it is possible that some of the conversion of NH_4_^+^ to PON was attributable to free-living bacterioplankton that passed through the GFF filter and particle-associated bacteria in the original ocean water. To the degree that this occurred, it would have contributed to an elevated PON/Chl a ratio in enrichment experiments. Consumption of phytoplankton by microzooplankton would also be consistent with this observation.

Approximately 30% of Chl-*a* biomass sinks out of the euphotic zone in the Bay of Fundy [35], and the bulk sediment flux consists mostly of algal cells and fecal pellets. Curiously, this benthic community is relatively impoverished, with low rates of macrobenthic production. As right whales in the area have frequently been observed with mud on their heads and bodies while feeding [36], the bioturbation they facilitate could be another function that has come close to vanishing with the near extinction of these whales.

Our study complements recent efforts in the Southern Hemisphere and in the southern Gulf of Maine to quantify the impact of great whales on marine ecosystems [3, 4, 6]. In the Southern Ocean, the iron content of whale feces is orders of magnitude higher than the background levels of Antarctic seawater [5]. Such high levels of iron in the surface layer could act as a fertilizer, and the krill-baleen whale system likely played a crucial role in the iron cycle of the Southern Hemisphere, especially before commercial hunting depleted many populations of whales.

In the North Atlantic, right whales appeared to be close to extinction since the 1930s [37]. Spatially explicit estimates suggest that there were 9,075 to 21,328 right whales in the North Atlantic before commercial whaling [38]. At that time, foraging whales and surface-active groups would have been more common, and perhaps larger in size and more extensive in duration—field observations indicate that the longer a SAG remains active, the larger it tends to be, as more whales approach the group over time [12]. This spatial and temporal cohesion suggests that SAGs can act like whale pump hotspots. Because these groups are highly visible and dynamic, they can also be useful for researchers. Researchers in the Auckland Islands relied on breeding aggregations to collect feces from southern right whales for micronutrient analysis [39], and earlier studies in the Bay of Fundy used samples collected during right whale courtship activity or when whales fluked up to dive [40].

The right whale population in the northwest Atlantic is comprised of approximately 450 individuals, and right whales are nearly extinct in the eastern North Atlantic. Naturally, their ecological impact has been greatly reduced since their populations were reduced from possibly more than 20,000. Before whaling and the rise in atmospheric deposition, right whales and other mysticetes played an important role in enhancing nitrogen and phosphorous on a local and global scale [4, 16]. Production of phytoplankton stocks that support copepods and other prey consumed by whales will benefit most immediately from the release of excreta in nutrient-limited waters, especially in the summer [4].

If efforts to restore populations of this once economically and ecologically important species are successful, the right whale’s influence on ecosystem processes might increase throughout its range: not only on its summer feeding areas of the northern Gulf of Maine and southwestern Scotian Shelf, but also in the winter calving grounds off Florida and Georgia and spring feeding areas along the Great South Channel and Cape Cod Bay. As numbers rise, we are sure to learn new ecological roles for this once common species. As consumers of copepods and other phytoplankton grazers, right whales could reduce grazing pressure on planktonic microalgae while adding nutrients through the whale pump; such a tritrophic mutualism has been proposed for seabirds (such as albatrosses, petrels, and shearwaters) and phytoplankton in the Southern Hemisphere [28]. Understanding the foraging behavior of right whales will not only help inform management of this endangered whale but also increase our understanding of vertical and horizontal nutrient transfer by this species.

It should be noted that major environmental changes appear to be underway in our study area. Sea surface temperatures in the Gulf of Maine have increased faster than 99% of the global ocean [41], and data collected by tags on basking sharks in 2012, the year following our study, indicated that the Bay of Fundy has undergone rapid subsurface warming [42]. There have been fewer right whales observed in the bay in recent years, perhaps a result of this temperature shift and changes in primary productivity and copepod abundances. Whether right whales will continue to thrive in this area, or discover new feeding grounds, remains to be seen.
